# Genetic, Clinical and Neuroradiological Spectrum of MED-Related Disorders: An Updated Review

**DOI:** 10.3390/genes16121444

**Published:** 2025-12-02

**Authors:** Alessandro Fazio, Roberta Leonardi, Lorenzo Aliotta, Manuela Lo Bianco, Gennaro Anastasio, Giuseppe Messina, Corrado Spatola, Pietro Valerio Foti, Stefano Palmucci, Antonio Basile, Martino Ruggieri, Emanuele David

**Affiliations:** 1 Department of Medical Surgical Sciences and Advanced Technologies “GF Ingrassia”, University Hospital Policlinic “G. Rodolico-San Marco”, 95125 Catania, Italy; lore.aliotta@gmail.com (L.A.); g_messina@me.com (G.M.); cor_spatola@hotmail.com (C.S.); pietrofoti@hotmail.com (P.V.F.); spalmucci@unict.it (S.P.); basile.antonello73@gmail.com (A.B.); 2 Neonatal Intensive Care Unit (NICU), A.O.U. Policlinico “G. Rodolico-San Marco”, P.O. “G. Rodolico”-University of Catania, 95123 Catania, Italy; leonardi.roberta@outlook.it; 3 Postgraduate Residency Program in Pediatrics, Department of Clinical and Experimental Medicine, University of Catania, 95123 Catania, Italy; 4 Unit of Pediatric Clinic, A.O.U. Policlinico “G. Rodolico-San Marco”, P.O. “G. Rodolico”-University of Catania, 95123 Catania, Italy; lobianco.manuela@gmail.com (M.L.B.); m.ruggieri@unict.it (M.R.); 5 Department of Educational Sciences, University of Catania, Via Teatro Greco, 95124 Catania, Italy; gennaro.anastasio@phd.unict.it; 6 National Center for Rare Diseases, Istituto Superiore di Sanità, Viale Regina Elena, 00161 Rome, Italy; 7 Department of Translational and Precision Medicine, Sapienza University of Rome, 00185 Rome, Italy

**Keywords:** genetic variants, MED complex, MED subunits, neurodevelopmental disorders, neuroimaging, rare diseases, transcriptional regulation

## Abstract

**Background/Objectives:** The Mediator (MED) complex is an essential regulator of RNA polymerase II transcription. There is increasing evidence that pathogenic variants in several MED subunits are the cause of neurodegenerative and neurodevelopmental phenotypes, collectively referred to as “MEDopathies”. This review aims to summarize current knowledge on the genetic basis, clinical manifestations, and neuroradiological features of MED-related disorders. **Methods:** We undertook a narrative synthesis of the literature focusing on the MED subunits most commonly associated with neurological disorders, including MED1, MED8, MED11, MED12/MED12L, MED13/MED13L, MED14, MED17, MED20, MED23, MED25, MED27, and CDK8. Sources included peer-reviewed genetic, clinical, and imaging studies, supplemented by relevant case reports and cohort analyses. In addition, representative facial phenotypes associated with selected MED variants (*MED11, MED12, MED13, MED13L, MED25*) were visualized for educational purposes using artificial intelligence-based image generation derived from standardized clinical descriptors. **Results**: All MEDopathies show converging clinical patterns: global developmental delay/intellectual disability, hypotonia, epilepsy, speech disorders, and behavioral comorbidity. Non-neurological involvement, such as craniofacial or cardiac anomalies, is subunit-specific. Neuroradiological features include callosal abnormalities (agenesis, thinning, dysmorphia), delayed or hypomyelination, progressive cerebral and cerebellar atrophy, basal ganglia signaling changes, pontine hypoplasia, and, in *MED27* deficiency, a “hot cross bun” sign. Gene-specific constellations emphasize catastrophic infantile progression (*MED11*), X-linked syndromes with callosal defects (*MED12/MED12L*), language-dominant phenotypes (*MED13*), and syndromic intellectual disability with systemic features (*MED13L*). **Conclusions**: The growing spectrum of MEDopathies argues for their recognition as a unified nosological group with overlapping clinical and radiological signatures. Characteristic MRI constellations may serve as diagnostic clues and guide targeted molecular testing. Future directions include longitudinal imaging to describe disease progression and the integration of genomic data with curated clinical radiological datasets to refine genotype-phenotype correlations.

## 1. Introduction

The Mediator complex (MED) stands at the forefront of gene expression regulation in eukaryotic organisms, orchestrating intricate molecular interactions crucial for cellular function. Its involvement extends beyond the realm of basic transcriptional control, encompassing pivotal roles in various biological processes, including those implicated in neurological diseases. This multiprotein complex has emerged as a key player in the intricate landscape of neurological disorders, ranging from acute conditions like stroke to chronic neurodegenerative disorders [1]. The intricate network of MED subunits has been implicated in a spectrum of genetic diseases, particularly those affecting the nervous system, with mutations in these subunits identified as contributors to a range of neurological alterations, thereby highlighting the complex interplay between molecular dysregulation within the MED complex and the manifestation of clinically relevant neurological pathologies, whose significance extends beyond a mere association to encompass active roles in brain adaptation and cognitive mechanisms, especially in response to various forms of neural deterioration. This unique role positions MED as a potential focal point for unraveling the underlying molecular mechanisms of neurological diseases and devising targeted therapeutic strategies [1]. Crucially, the intersection of genetics, clinic, and radiology in the context of MED-related pathologies opens a fascinating avenue of exploration. Beyond unraveling the molecular basis of these diseases, the possibility of identifying distinct radiological patterns associated with MED mutations offers a novel dimension to diagnostic precision. In this literature review, we delve into the multifaceted roles of MED in neurological diseases, emphasizing the potential for developing specific radiological markers that could enhance our ability to diagnose and understand the diverse clinical manifestations associated with MED mutations. Through this exploration, we aim to bridge the gap between genetic understanding and clinical application, paving the way for a more nuanced and targeted approach to the diagnosis of MED-related neurological pathologies.

## 2. Rationale, Scope, and Structural Roadmap

This narrative review was undertaken to provide a comprehensive, multidisciplinary synthesis of the most current findings on the MED complex and its associated diseases. These rare disorders, often scattered across diverse case reports and specialized studies, can be difficult for clinicians and researchers to access and interpret. By bringing together the existing evidence on genetic mutations, clinical manifestations, and radiological features, this review aims to create a coherent overview that makes the complex field of MED-related diseases more approachable. As a non-systematic review, it is written in an accessible and readable format, allowing the reader to consider the topic broadly while appreciating its detailed nuances. By clearly presenting the structure of this review, the authors have organized a complex and diverse body of knowledge, highlighting the interplay between molecular mechanisms, clinical features, and radiological findings. After this introduction, the review first explores the molecular composition of the MED complex, providing essential background for understanding its role in human disease. The subsequent sections focus on each MED subunit implicated in neurological disorders, integrating evidence from key studies that have described the relevant gene mutations, clinical presentations, and associated pathologies. For each subunit, the discussion draws upon the current literature to summarize the genetic alterations, characteristic clinical manifestations, and notable imaging features, providing a comprehensive synthesis of what is known to date. Furthermore, with the aim of making visible the typical clinical facial characteristics described in certain specific mutations, Artificial Intelligence (AI), particularly ChatGPT 5, was used to recreate these characteristics in illustrations that are shown in the text. The specific details regarding the procedure we used are described in Appendix A and Supplementary Materials.

## 3. Composition and Structure of MED Complex

**Figure 1:**
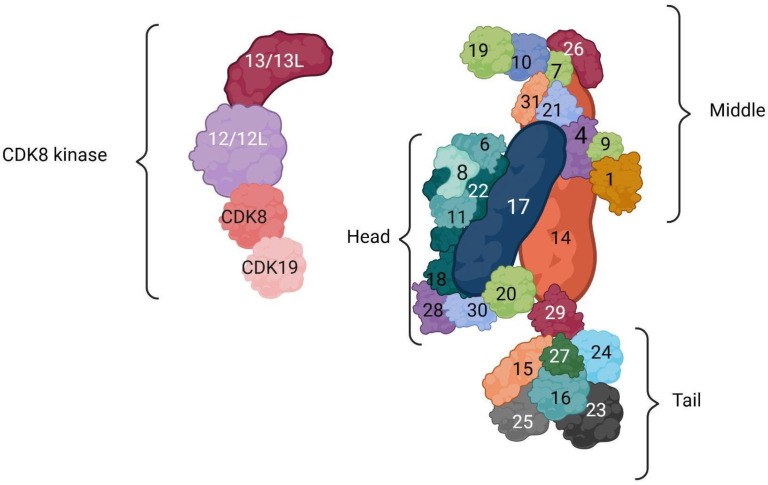
**MED complex structure.** This figure was created with biorender.com.

The MED complex, a highly conserved multi-component entity, plays a pivotal role in the regulation of gene transcription. This complex is organized into distinct sections: the head, middle, and tail modules (Figure 1).

The head and middle modules have a direct connection with RNA polymerase II (Pol II), contrasting with the tail module, which does not associate with Pol II. The human MED, comprising 26 tightly interlinked subunits, displays a higher level of functional integration, especially when compared to its yeast counterpart [2]. Key components, MED14 and MED17, extend across the complex’s length, contributing to a structural core made up of an α-helical bundle that includes nine subunits [3]. This structure acts as a critical framework. Additionally, the MED’s function is significantly modified by the kinase module, a detachable yet stable component that inhibits the complex’s interaction with Pol II. The inherent structural flexibility and disorder within the MED enable it to function as a central coordinator for various inputs, facilitating specific regulatory responses. Differences in subunit makeup, sequences, and structural organization distinguish the MED complexes across species, from yeast to humans. Studies involving genetic manipulation have highlighted the modular independence within the yeast complex, particularly the ‘head’ and ‘middle’ modules’ direct interactions with Pol II. This modular concept extends to mammalian Mediators, implying a preserved architectural principle. MED14 and MED17 subunits, found across different species, bridge the entire complex, linking the three modules. They are integral to an α-helical bundle involving nine subunits, laying down a substantial structural foundation [3]. The human bundle is notably larger than its yeast equivalent, supporting an expanded ‘tail’ module capable of processing a broader range of regulatory signals and facilitating structural adjustments. The ‘tail’ module’s adaptability, more so than the ‘head’ and ‘middle’ modules, allows it to assume various structural configurations [4]. The kinase module, consisting of four subunits, attaches to and detaches from the MED, forming a stable entity within cells and possibly operating independently of the MED in human cells [3]. This module, identifiable as a distinct unit in human cells, undergoes selective degradation in yeast under nutrient scarcity. Its components—CDK8, cyclin C, MED12, and MED13—are preserved from yeast to humans, despite variations in sequences and sizes. In higher eukaryotes, the Mediator complex contains a distinct regulatory assembly known as the CDK8 kinase module (CKM), which can reversibly associate with the core Mediator to modulate transcriptional activity. This module is composed of CDK8 or its paralog CDK19, cyclin C, and the two subunits MED12 and MED13, each of which has a paralog—MED12L and MED13L, respectively. MED12 and MED12L, as well as MED13 and MED13L, are paralogous genes that encode components of the kinase module of the Mediator complex. To be more precise, *MED12* is located on Xq13.1, while *MED12L* is on 3q25.1. Likewise, *MED13* maps to 17q23.2, and *MED13L* to 12q24.21. As discussed by Soutourina (2017), the emergence of MED12L and MED13L in vertebrates resulted from gene duplication events, producing paralogous proteins with high sequence similarity and conserved regulatory functions. The CDK8 module, through the interplay of these subunits, associates transiently with the Mediator core, providing an additional level of control over transcription initiation by influencing pre-initiation complex formation and promoter engagement [5].

While the yeast kinase module’s structure is known, detailed insights into the human variant remain elusive [3]. The human kinase module, roughly 600 kDa in size, significantly influences MED’s role by blocking its connection with Pol II and excluding the MED26 subunit, exclusive to metazoans, thus forming a 29-subunit CDK–MED complex [6].

**Figure 2:**
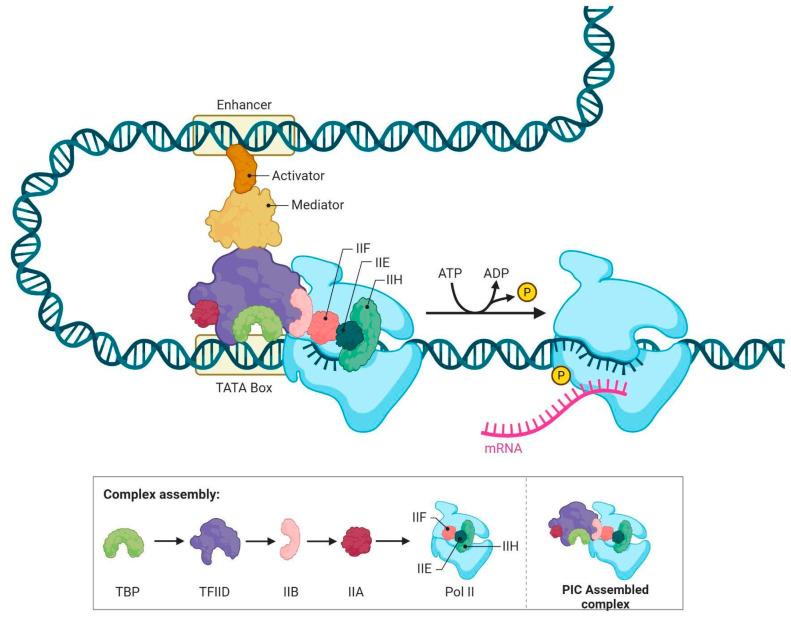
The Mediator complex serves as a molecular bridge, linking transcriptional activators bound to enhancer regions with the basal transcription machinery assembled at the promoter. During chromatin looping, Mediator facilitates the recruitment and stabilization of general transcription factors (TFIIA, TFIIB, TFIID, TFIIE, TFIIF, TFIIH) and RNA polymerase II, promoting the formation of the pre-initiation complex (PIC) on the TATA box. CDK7, a subunit of TFIIH, phosphorylates the C-terminal domain (CTD) of RNA polymerase II, triggering promoter escape and the transition to productive elongation. This dynamic process ensures precise regulation of gene expression, integrating regulatory signals from enhancer-bound transcription factors into transcriptional output. This figure was created with biorender.com.

A schematic representation of Mediator complex function during RNA polymerase II transcription initiation has been shown in Figure 2.

In the following sections, we will examine each subunit of the MED complex, analyzing numerous studies from the literature. This analysis will encompass the mutations involved, as well as the clinical and radiological profiles associated with these genetic variations.

## 4. *MED1* (MIM *604311) [7]

*MED1* [7], also recognized as *TRAP220* (thyroid hormone receptor-associated protein), functions as a pivotal enhancer for nuclear receptors, playing an essential role in both the growth and differentiation processes within the central nervous system [8]. The lack of *MED1* in mouse models has led to embryonic fatality and abnormalities in the neural tube. Throughout the stages of embryonic growth, the presence of *MED1* was noted across different cerebral regions, showing notable levels of expression in certain areas [1]. A recent animal study suggested the role of *MED1* neuronal expression in neuroplastic changes, particularly showing that thalamic hemorrhage elicits a significant increase in hippocampal *MED1* and Tropomyosin receptor kinase B (TrkB) expression, accompanied by a decrease in Brain-derived neurotrophic factor (BDNF) levels, all of which were restored in rodent models by a combined N-palmitoylethanolamide and luteolin treatment [9]. M. Leonard et al. have identified MED subunit 1 (MED1) as an estrogen receptor (ER) coactivator, a unique and tissue-specific cofactor mediating breast cancer metastasis and treatment resistance. *MED1* is overexpressed in over 50% of human breast cancer cases and co-amplifies with another critical breast cancer gene, the HER2 tyrosine kinase receptor [10]. Clinically, *MED1* expression is highly correlated with poor disease-free survival in breast cancer patients, and recent studies have reported an increased frequency of MED1 mutations in circulating tumor cells of patients after treatment [11].

## 5. *MED8* (MIM *607956) [12]

*MED8* is responsible for producing a protein of the MED complex, initially discovered in *S. cerevisiae*, essential for the initiation of transcription [13]. The possible implication of *MED8* in neurological diseases has been suggested by its relationship with the Seizure Threshold 2 gene (*SZT2*) (MIM *615463) [14], whose mutations are related to epileptic encephalopathy and intellectual disability [15]. In fact, some preclinical studies revealed that the *MED8* gene and *SZT2* have a similar pattern of expression, possibly sharing a promoter [16]. Notably, the *SZT2* gene is situated in a highly conserved head-to-head configuration with MED8 in mammals [17]. Although no direct protein interaction between *MED8* and *SZT2* has been observed in co-immunoprecipitation experiments in transiently transfected COS-7 cells, the proximity of their promoter sequences suggests potential regulatory connections. *SZT2* demonstrates widespread transcription in various tissues, with a notable expression in the brain, including during embryonic development [17]. Despite the unknown biological function of *SZT2/MED8*, the significant conservation and impact on epileptogenesis suggest its potential role in the central nervous system. While *MED8*’s role in neurological diseases has been suggested in preclinical studies, the specific mechanisms and clinical consequences underlying the involvement of *SZT2/MED8*’s interaction remain areas for further investigation [17].

## 6. *MED11* (MIM *612383) [18]

**Figure 3:**
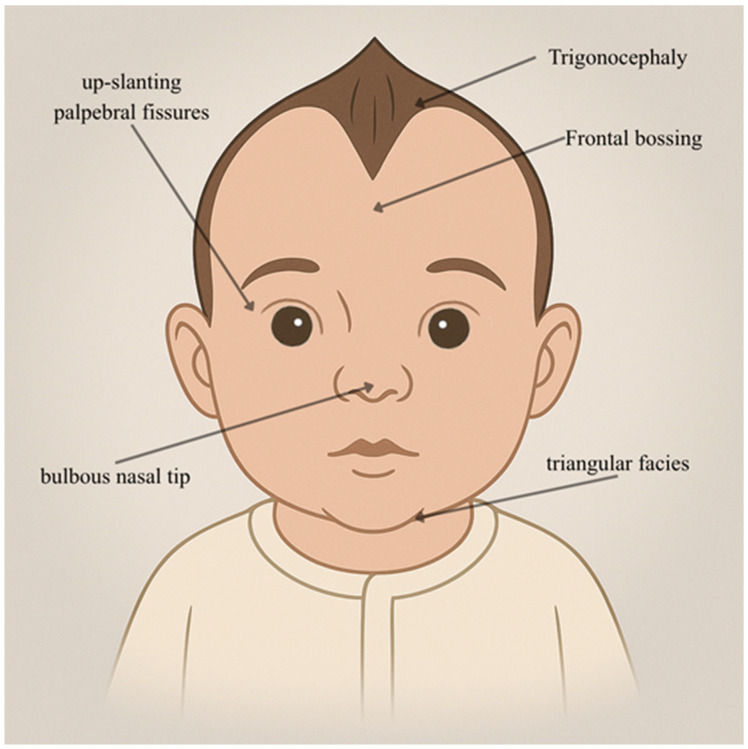
**Facies characteristics in *MED11* mutations** (c.325C>T; p.Arg109Ter). This figure shows a newborn male with a triangular cranial shape (trigonocephaly), frontal bossing, triangular facies, and a bulbous nasal tip. This image was created using Artificial Intelligence (AI), particularly ChatGPT 5. Details regarding the exact procedure are contained in the Supplementary Materials.

*MED11*, part of the MED complex, plays a crucial role in gene expression regulation. The MED complex acts as a bridge between transcription factors and transcriptional machinery, facilitating the initiation and regulation of gene transcription [19]. MED11, as a subunit within the complex, contributes to its stability, ensuring proper functioning in the control of gene expression. Mutations in *MED11* have been associated with neurodegenerative disorders, highlighting its significance in maintaining cellular homeostasis [20]. This brief introduction draws insights from the study conducted by E. Calì et al. in 2022, where a recurrent homozygous truncating variant in *MED11* was identified, leading to a novel autosomal recessive neurodegenerative disorder [20]. This international observational exome/genome-sequencing study, complemented by in vitro functional assays and in vivo zebrafish knockouts, characterized a recurrent homozygous *MED11* truncating variant (c.325C>T; p.Arg109Ter) in seven patients from five unrelated families. Despite *MED11* truncation, protein and transcript levels resembled healthy controls, suggesting escape from nonsense-mediated decay. Computational analysis revealed variant-induced disruptions among MED11, MED28, and MED30C subunits, emphasizing the role of the MED11 C-terminal region in forming a hydrophobic structure. The findings provided crucial molecular insights into the disorder’s genetic basis. Clinically, affected individuals exhibited severe features, including distinctive craniofacial characteristics (as shown in Figure 3, trigonocephaly, frontal bossing, triangular facies with up-slanting palpebral fissures, and bulbous nasal tip), congenital microcephaly, and early-onset respiratory distress.

Four individuals succumbed to cardiorespiratory insufficiency between 24 h and 10 days of life, and two required prolonged assisted ventilation into childhood. Global developmental delay with failure to achieve any milestones was universal; all patients exhibited neonatal-onset refractory myoclonic seizures and severe encephalopathic EEG patterns, but also axial hypotonia with appendicular hypertonia. Additional features comprised impaired hearing, vision issues, and CNS atrophy. These symptoms are associated with severely progressive diffuse central nervous system atrophy/degeneration, starting prenatally, and leading to premature death in early infancy or childhood. Magnetic resonance imaging (MRI) was conducted on the patients enrolled in the study, revealing radiological signs summarized in Table 1 [20].

## 7. *MED12* (MIM *300188) [21]

*MED12* plays a crucial role in controlling gene transcription within the CDK8 subcomplex, also including CDK8, cyclin C, and MED13. Recent studies have shown that, unlike previous assumptions about CDK8’s kinase activity being key for repressing transcription [22].

**Figure 4:**
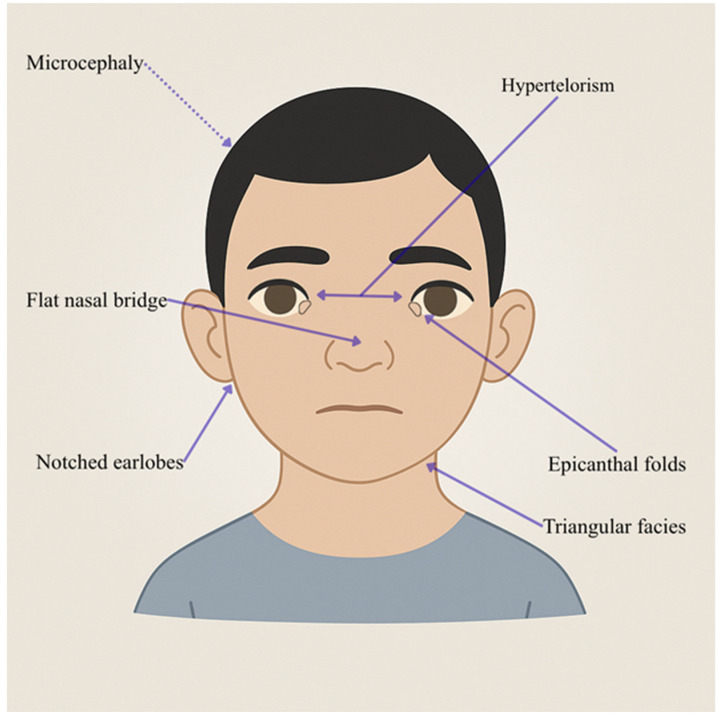
Facies characteristics in *MED12* mutations (p.Asn1007Ser and related variants). This figure shows a male child (6 years) with triangular facies, a flat nasal bridge, epicanthal folds, hypertelorism, and notched earlobes. This image was created using AI, particularly ChatGPT 5. Details regarding the exact procedure are contained in the Supplementary Materials.

*MED12*, also referred to as *TRAP230*, has been identified as playing a critical role in the development of wing and eye disks in Drosophila melanogaster [23]. Research conducted by Kim et al. highlighted *MED12*’s importance in neural stem cells (NSCs), particularly its connections to the networks of “cell-cell interaction” and the “cell cycle.” The absence of *MED12* in NSCs led to an increase in cell adhesion, driven by the upregulation of adhesion genes like Syndecan 2, suggesting *MED12* serves as a negative regulator of NSC adhesion. Additionally, research has shown that the MED complex plays a key role in the development of neurons within the epithalamus [24]. Site-specific investigations have uncovered that mutations in *MED12* in zebrafish result in changes in habenular and parapineal neurons, affecting FGF signaling and the regulation of *TBX2B* gene expression, which is vital for the differentiation of parapineal cells [25]. Disorders arising from variations in *MED12* are identified as conditions associated with *MED12*. Positioned on Xq13.1, *MED12* exhibits a prevalence in male patients inheriting the missense variant from unaffected carrier females [26]. Hemizygous *MED12* variations are linked to several X-linked developmental syndromes, including Opitz-Kaveggia syndrome (FG syndrome) (MIM #305450) [27], Lujan-Fryns syndrome (MIM #309520) [28], and X-linked Ohdo syndrome (MIM #300895) [29], alongside non-X-linked intellectual disability (ID) syndrome [30]. These syndromes manifest symptomatic parallels, encompassing intellectual disability, hypotonia, macrocephaly, hyperactivity, anomalies in the corpus callosum, behavioral irregularities, and distinctive facial features (Figure 4).

Genetic, clinical, and radiological characteristics of these X-linked disorders related to *MED12* are summarized in Table 2 [31].

Recently, females with *MED12* variants have also been identified [32]. Surprisingly, in addition to missense variants, nonsense and frameshift variants have been found. Li et al. describe a cohort of seven female patients with Hardikar syndrome (MIM #301068) [33], a rare syndrome characterized by multiple congenital anomalies, including facial clefts, retinitis pigmentosa, biliary anomalies, and intestinal malrotation [32]. Namik Kaja et al. in their study aimed to understand a novel genomic disorder with profound phenotypic consequences, most notably global developmental delay, autism, psychosis, and anorexia nervosa. They assessed individuals from an extended family affected by a maternal-related condition using the Childhood Autism Rating Scale (CARS), Vineland Adaptive Scales, MRI, magnetic resonance spectroscopy (MRS), electroencephalography (EEG), electromyography (EMG), muscle biopsy, and various genetic tests [34]. They identified a new Xq12–q13.3 duplication in three patients within an extended family, notably affecting clinically asymptomatic mothers who exhibited a clear preference for the non-duplicated X chromosome. Comprehensive genetic analysis revealed significant modifications in gene expression and pathways, aligning with previous studies on autism spectrum disorders (ASD). This chromosomal aberration, Xq12–q13.3 duplication, represents a novel predisposition for global developmental delay and autism, potentially mediated by heightened gene dosage, including *NLGN3, OPHN1, AR, EFNB1, TAF1, GJB1*, and *MED12* [34]. Table 3 provides a comprehensive summary of the clinical, neurological, radiological, and therapeutic features of the three patients, highlighting key patterns and distinctive characteristics.

In another study, Paolo Frontera et al. identified a distinct clinical phenotype characterized by minor anomalies, severe intellectual disability (ID), and absent language in both male and female family members carrying a frameshift mutation in the *MED12* gene. Three individuals within the same family were examined: they had compromised language abilities to the point of absent language, but normal brain MRI, EEG, echocardiography, and standard blood tests. Whole exome sequencing (WES) revealed a novel *MED12* mutation (c.2312T>C). This finding suggests the existence of a fourth clinical phenotype associated with *MED12*, characterized by severe ID, absent or limited language, and milder clinical manifestations in heterozygotes [35]. In the research conducted by Ying Jiang et al., the study highlights the association between *MED12* gene mutations and agenesis of the corpus callosum. Two cases were presented: in the first case, a 21-year-old G1P0 female exhibited complete agenesis of the corpus callosum, confirmed by fetal MRI, and subsequently delivered a female fetus at 31 weeks. The second case involved a 33-year-old G1P0 female with fetal ultrasound at 26 weeks, revealing agenesis of the corpus callosum. Subsequent MRI at 27 weeks confirmed complete callosal agenesis along with a Dandy-Walker variant malformation, and this pregnancy resulted in a live birth. WES identified a homozygous missense mutation in exon 27 of the *MED12* gene on the X chromosome and a de novo heterozygous missense mutation in intron 1 of the *EFNB1* gene on the X chromosome. These findings contribute to our understanding of the genetic basis of corpus callosum abnormalities and expand the spectrum of associated *MED12* gene mutations [36].

## 8. *MED12L* (MIM *611318) [37]

*MED12L* is a subunit of the kinase module, which is one of the four subcomplexes of the MED complex [22]. Nizon et al. recruited seven individuals with *MED12L* variants via genetic analysis [38]. All subjects exhibited intellectual disability and/or developmental delay, along with additional features such as autism spectrum disorder and aggressive behavior. Variants comprised deletions, duplications, nonsense mutations, frameshifts, and splicing alterations. The cohort, consisting of four males and three females, predominantly presents intellectual disability/developmental delay. Common traits include abnormal behaviors, frequently associated with autism spectrum disorder, attention deficit hyperactivity disorder, sleep disorders, and hypotonia [38]. Genetic analysis revealed duplications or deletions within the *MED12L* gene, with evidence of de novo variants in some individuals. Functional analysis confirmed a significant disruption in RNA synthesis in two individuals with these variants, suggesting a haploinsufficiency mechanism leading to a transcriptional defect. Despite an absence of a clear genotype-phenotype correlation, similarities emerged with conditions linked to *MED12*, *MED13*, and *MED13L*. Corpus callosum abnormalities were found to be prevalent in both *MED12* and *MED12L* [38]. The involvement of *MED12L* in human pathologies through transcriptional defects, like other subunits of the MED complex, could outline a shared clinical model between conditions associated with transcriptional defects linked to the MED complex.

## 9. *MED13* (MIM *603808) [39]

**Figure 5:**
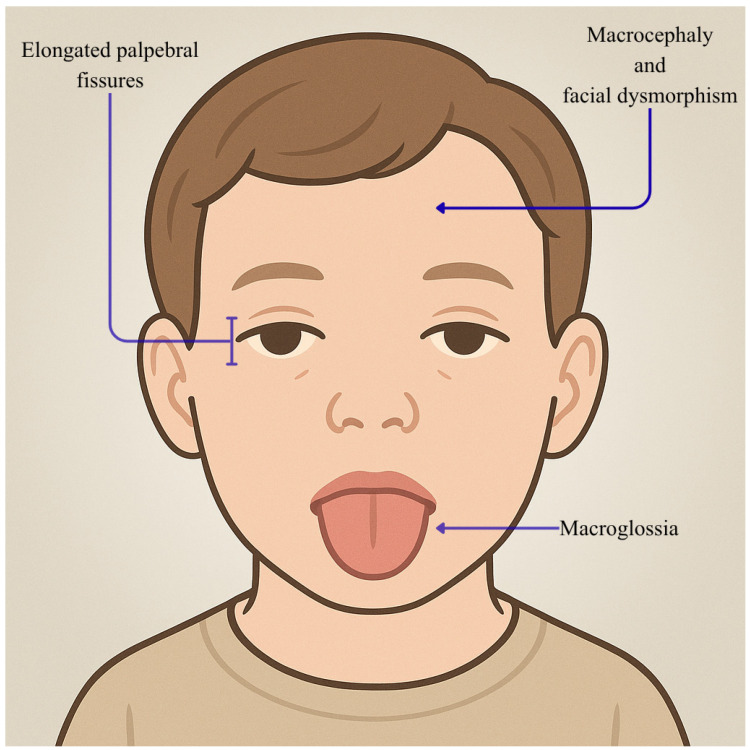
Facies characteristics in *MED13* mutations (p.Pro327Ser and related variants). This figure shows a male child (5 years) showing elongated palpebral fissures, macroglossia, macrocephaly, and atypical facial proportions. This image was created using AI, particularly ChatGPT 5. Details regarding the exact procedure are contained in the Supplementary Materials.

*MED13* gene, part of the CDK8 kinase module, plays a crucial role in Pol II transcription initiation by reversibly binding to the MED complex [22]. Variations in genes encoding MED subunits, such as *MED13L*—a counterpart of *MED13*—have been linked to developmental and intellectual disabilities, notably affecting language. Research by L. Snijders Blok et al. on a cohort of 13 individuals revealed 11 novel mutations in *MED13*, with one case of inheritance [40]. These individuals primarily exhibited intellectual and developmental delays, including language impairments. Other common traits involved autism, attention-deficit hyperactivity disorder (ADHD), optic nerve anomalies, Duane anomaly, hypotonia, congenital heart defects, and facial dysmorphisms. An MRI scan of a patient showed a minor left occipital lobe anomaly, but this did not correlate with their seizure characteristics. Other subjects either lacked MRI data or presented no significant findings, aside from one instance of mild frontal atrophy. Visual issues were rare, though optic nerve irregularities appeared in three cases, including paleness, papilledema, and retinal atrophy near the optic disks. Genetic analysis identified truncating, missense mutations, and a single amino acid deletion within *MED13*, with a concentration of non-truncating mutations at the protein’s ends. Significantly, mutations affecting amino acids p.Thr326 and p.Pro327 disrupt *MED13*’s ubiquitination and degradation process, highlighting a specific molecular pathology pathway. Laura De Nardi et al. documented the case of a 13-year-old girl previously diagnosed with Kabuki syndrome (KS) (MIM #147920) [41], a rare disorder characterized by mild to moderate intellectual disability and developmental delay associated with a typical facial gestalt, but lacking mutations in classical KS genes [42]. WES revealed a de novo missense mutation in *MED13* (c.C979T; p.Pro327Ser), a variant previously implicated in a newly identified neurological disorder. Currently, only one case of epilepsy linked to a *MED13* mutation has been documented [43]. This individual displayed drug-resistant generalized epilepsy from infancy, characterized by various seizure types. Developmental and epileptic encephalopathies (DEEs) represent neurological disorders where developmental delays, epilepsy, and cognitive and behavioral issues arise from severe brain disorders [44]. Both epilepsy and the underlying disease contribute to developmental delays. Trivisano’s study presents the electroclinical features of a patient with a de novo *MED13* mutation, displaying severe developmental delay and epilepsy, including focal seizures and infantile spasms [43]. The clinical case involves a 24-month-old boy with epilepsy onset at 3 months old, featuring apnea, limb hypertonia, and multifocal epileptiform abnormalities on EEG. Epileptic encephalopathy emerged at 10 months old, with subsequent epileptic spasms, and has been treated with midazolam, vigabatrin, and phenytoin; ACTH for spasms. The patient exhibited severe hypotonia and dysmorphic features, including microcephaly and elongated eyelids. Brain MRI revealed abnormalities, including a dysmorphic corpus callosum. WES identified a novel *MED13* gene mutation: this demonstrates the wide phenotypic variability of MED13-related disorders [38]. Initially, *MED13* mutations emerged within a significant cohort of ASD-diagnosed individuals identified through whole-genome sequencing (WGS). Subsequently, Snijders-Block et al. documented 13 patients exhibiting developmental delay and/or variable intellectual disability (ID), accompanied by speech disorders, ASD, ADHD, eye abnormalities, and mild dysmorphic features [40]. Later, a de novo *MED13* mutation was detected in a patient demonstrating clinical features resembling those of Kabuki syndrome [42]. Rogers et al. described global developmental delay and global developmental delay, marked facial dysmorphism, macroglossia, short stature, and macrocephaly in an additional patient [45]. Altogether, 19 cases of *MED13* mutations have been cataloged. Notably, among these cases, only one patient presented with epilepsy, specifically drug-resistant myoclonic-atonic epilepsy (MAE) [43]. However, the epileptic profile of the patient in Trivisano’s study, who exhibited earlier onset and more severe symptoms, including infantile spasms and focal seizures. Moreover, while the patient with MAE displayed borderline cognitive development, other patients presented with severe developmental delay and pronounced hypotonia [43]. As illustrated in Figure 5, common features observed in previously reported cases include microcephaly, developmental delay, hypotonia, corpus callosum (CC) abnormalities, deafness, and retinal atrophy.

## 10. *MED13L* (MIM *608771) [46]

**Figure 6:**
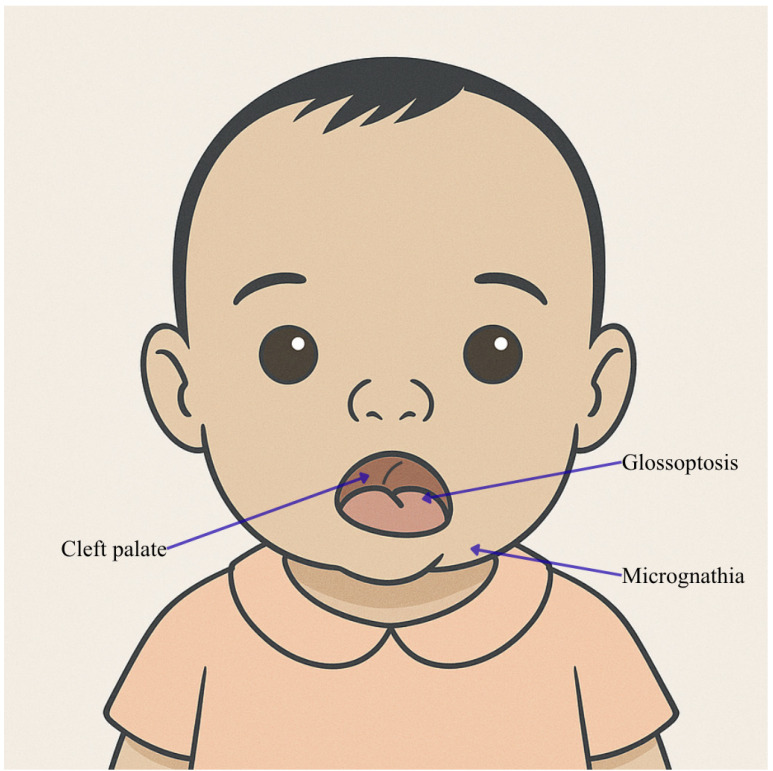
Facies characteristics in *MED13L* mutations (variants associated with Pierre Robin sequence). Newborn male with micrognathia, glossoptosis, and cleft palate. This image was created using AI, particularly ChatGPT 5. Details regarding the exact procedure are contained in the Supplementary Materials.

*MED13L* encodes a subunit of the MED complex, functioning as a transcriptional coactivator for all RNA polymerase II-dependent genes [47]. Structural variations and gene mutations are involved in a syndrome characterized by craniofacial abnormalities and intellectual disabilities with or without cardiac defects [48]. Clinically, the facial features of the affected patients are illustrated in Figure 6a,b. In the past years, mutations in this gene have been detected in over 70 patients. Asadollahi et al. focused on individuals with *MED13L* mutations, presenting with syndromic intellectual disability without complex congenital heart defects [49]. These variations, including *MED13L* and the adjacent gene *MAP1LC3B2*, lead to out-of-frame deletions that correlate with a subtler phenotype. The radiological findings highlighted include delayed cortical myelination, short and thick corpus callosum, cysts from cavum vergae, arachnoid cysts, and periventricular small round signal alterations suggestive of myelination disorder. Additionally, Gordon et al. by analyzing the entire exome of two unrelated patients affected by Pierre Robin Syndrome (PRS) (MIM #261800) [50], an essential splice site mutation in *MED13L* was discovered in one, and an intragenic deletion in the other: both mutations, disrupting a splicing site of the MED complex, are crucial for transcription regulation [51]. Radiological findings in these patients included periventricular white matter anomalies in the first patient and a thin corpus callosum, ventricular dilatation, and subcortical white matter anomalies in the second. These findings expand the clinical spectrum of *MED13L*-associated disorders to include PRS, emphasizing the necessity of considering *MED13L* mutations in the differential diagnosis of syndromic PRS.

One study summarizes findings from a study involving seven patients with protein-truncating variants in the *MED13L* gene, along with a family carrying a large duplication of *MED13L*. All individuals with these variants showed intellectual disability and shared common facial features. Interestingly, none of the patients had complex congenital heart defects, challenging previous beliefs about MED13L’s role in heart disease. The study suggests a broader spectrum of the *MED13L* syndrome, where cardiac and autism features may or may not be present for diagnosis [52]. Recently, two *MED13L* variants have been classified as pathogenic nonsense mutations; one of these variants was found in siblings [53]. Most recently, the first case of paternal germinal mosaicism for a missense *MED13L* variant causing intellectual disabilities and distinctive facial features, with or without cardiac defects syndrome, was identified in one of the father’s children, being the cause of IDs and facial dysmorphia in the other [54].

## 11. *MED14* (MIM *300182) [55]

Lou et al. examined the roles of *MED14* and *Brg1*, components of the MED complex and BAF complexes, respectively, in the differentiation of skeletogenic neural crest cells in zebrafish embryos [56]. The researchers discovered that mutations in either *MED14 or BRG1* resulted in a spectrum of defects related to neural crest cell differentiation, particularly affecting the development of jaw cartilage. Despite normal neural crest cell specification and migration, the mutants exhibited significant issues in terminal differentiation processes at their target sites. Experiments, including transplantation analyses, indicated that *MED14* and *Brg1* function in a cell-autonomous manner within neural crest cells to facilitate their differentiation. Additionally, the study revealed strong genetic interactions between *MED14* and *Brg1*, suggesting a collaborative role in neural crest development [56]. Therefore, this observation would clarify the nature of the defects in some craniofacial anomalies.

## 12. *MED16* (MIM *604062) [57]

**Figure 7:**
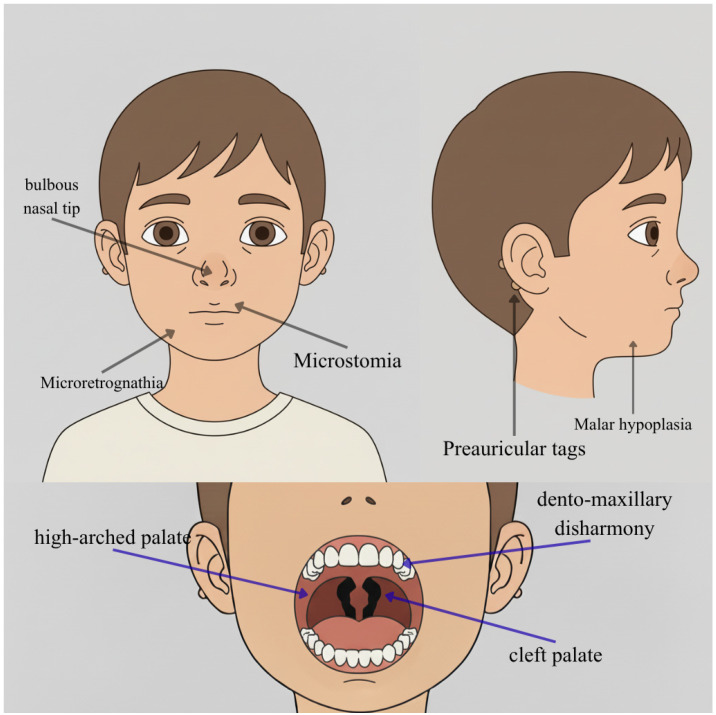
Facies characteristics in *MED16* mutations. This figure shows a male child (6 years) showing microretrognathia, malar hypoplasia, bulbous nasal tip, thin nostril wings, and a short columella. This image was created using AI, particularly ChatGPT 5. Details regarding the exact procedure are contained in the Supplementary Materials.

Biallelic pathogenic variants in *MED16* have recently been linked to a distinctive autosomal recessive multiple congenital anomaly syndrome characterized by neurodevelopmental impairment and multisystem involvement. Guillouet C. et al. have recently published the principal study on this specific mutation, reporting the largest reported cohort, in which developmental delay or intellectual disability and severe speech impairment were nearly universal, often accompanied by feeding and respiratory difficulties in early life [58]. Additionally, diagnoses of ASD, ADHD, and epilepsy occurred in a minority of cases. As shown in Figure 7, craniofacial anomalies such as micrognathia, cleft palate, and preauricular tags, together with thumb hypoplasia or aplasia, brachydactyly, nail hypoplasia, and occasional polydactyly, defined a recognizable dysmorphic spectrum [58].

Congenital heart defects, most frequently tetralogy of Fallot, are a major recurrent feature, while visual and auditory impairment were also reported as common. Brain MRI abnormalities were reported in more than half of the evaluated individuals. The most consistent neuroradiological findings were corpus callosum abnormalities, ranging from posterior thinning/shortening to complete agenesis, and ventriculomegaly or enlarged lateral ventricles in several cases [58]. Genetic variants include both predicted loss-of-function alleles and missense changes clustering in conserved protein domains; functional studies indicate impaired nuclear localization of MED16 protein as a plausible pathogenic mechanism [58]. Given the combination of neurodevelopmental impairment, distinctive craniofacial features, limb anomalies, and a high prevalence of conotruncal cardiac defects (notably tetralogy of Fallot), *MED16* should be considered in the differential diagnosis for autosomal recessive MEDopathies and MCA syndromes. When *MED16* variants are identified, targeted clinical evaluations, including cardiac imaging, ophthalmological and audiological screening, and brain MRI, are indicated.

## 13. *MED17* (MIM *603810) [59]

The *MED17* gene encodes for RNA polymerase II transcription MED subunit 17, a co-activator crucial for initiating transcription by RNA polymerase II [60]. *MED17* mutations lead to autosomal recessive disorders like microcephaly, intellectual disability, epilepsy, and ataxia, as evidenced by various clinical cases [61]. In 2010, Kaufman et al. identified a distinct form of progressive postnatal microcephaly and severe developmental delay associated with cerebral and cerebellar atrophy in Israeli children of Caucasian descent, linked to a specific homozygous missense mutation in the *MED17* gene [62]. Brain MRI depicted extensive and pronounced cerebral and cerebellar atrophy, noticeable as early as 3 months old. Subsequent scans indicated inadequate myelination, along with cerebral and cerebellar atrophy, diminished thalamic size, and a slender brainstem. Further research has documented pathological alterations in *MED17*. Agostini et al. reported on two adult sisters with compound heterozygous *MED17* mutations, showing a milder phenotype characterized by progressive microcephaly, moderate cognitive impairment, and epilepsy [63]. Their MRI scans revealed slight cerebellar vermis shrinkage but no signs of cerebral atrophy. A. Fattal-Valevski et al. conducted an extensive characterization of a disease associated with the *MED17* gene. They conducted a medical records review to gather clinical, laboratory, and neuroimaging data from unrelated Caucasian-Jewish families diagnosed with the same homozygous *MED17* mutation [64]. This study unveiled a pattern of progressive microcephaly, severe developmental delays, and diverse neurological symptoms. Importantly, MRI scans revealed significant cerebral and cerebellar atrophy, along with specific alterations such as reduced white matter volume, delayed myelination, corpus callosum thinning, and pronounced ventral pons flattening. Rafiullah et al. investigated a consanguineous family with severe intellectual disability (ID), seizures, and progressive microcephaly. Brain MRI showed mild atrophy and myelination defects. WES identified a novel homozygous missense variant in *MED17*, with parental heterozygosity [65]. In contrast to prior findings regarding the Caucasian Jewish demographic [64], S. Hirabayashi and colleagues present the case of two unrelated brothers exhibiting nystagmus and sudden opisthotonic posture from early childhood, followed by developmental delay and significant choreiform movements accompanied by hypotonia [66]. The brother experienced mild microcephaly postnatally, while the sister’s brain MRI revealed slight delays in myelination, anterior horn dilation, and mild cerebellar atrophy. WES identified compound heterozygous mutations in the *MED17* gene in both siblings, inherited from their respective parents. In comparison to the cases analyzed by Kaufmann [62], these observations demonstrate milder clinical manifestations and slower disease progression, along with more restricted MRI abnormalities. Table 4 summarizes clinical, radiological, and genetic characteristics of literature-reported MED-17-related cases.

## 14. *MED20* (MIM *612915) [67]

*MED20*, an integral part of the head module of the MED complex, is crucial for the regulation of gene expression through RNA polymerase II, and its mutations are linked to movement disorders and neurodegeneration. Julia Vodopiutz and colleagues explored *MED20*’s influence on a specific, autosomal recessive disorder marked by early-onset spasticity, dystonia during childhood, and the gradual deterioration of the basal ganglia and brain (MIM #616145) [68,69]. This study aimed to pinpoint the genetic anomaly in affected siblings using genetic linkage and WES, expanding the search to six unrelated individuals for comprehensive mutation analysis in *MED20*. MRI scans indicated significant atrophy within the brain, modifications in the basal ganglia, a narrowed corpus callosum isthmus, and cerebellar reduction from a youthful age, progressing to more severe atrophy and basal ganglia hyperintensity in MRI findings by adolescence. Additionally, no lactate peak was observed in the spectroscopy of the basal ganglia and frontal white matter, along with a notable decrease in NAA levels [69]. However, these findings are notably constrained due to the data being sourced from a singular family unit. Therefore, for a more robust validation of the *MED20* mutation’s role in the condition, it will be essential to uncover similar biallelic mutations in *MED20* among patients who are not related but share comparable clinical manifestations.

## 15. *MED23* (MIM *605042) [70]

MED23 is a subunit of the MED complex, physically associates with the heterodimeric RNF20/40 E3-ligase complex to facilitate the monoubiquitylation of histone H2B on gene bodies of actively transcribed genes. Mutations in this gene are strongly associated with intellectual disability, even severe ones. In 2011, Hashimoto et al. documented a mutation (p. R617Q) in *MED23*, an essential part of the MED complex that plays a pivotal role in gene expression regulation, associated with autosomal recessive intellectual disability that does not present with syndromic features [71]. This particular mutation interferes with the activation of the JUN and FOS genes, which are crucial for the cellular response to serum mitogens, by impacting the interaction between the MED complex and transcription factors *TCF4* and *ELK1*. This disruption in transcription regulation mirrors the abnormalities seen in patients with neurological disorders stemming from mutations in various components of the MED complex or its interacting proteins. These findings underscore the critical importance of the MED complex in brain development and functioning, indicating that the altered expression of immediate early genes could be a common denominator in the pathology of cognitive impairments. A. Trehan et al. focused on two non-consanguineous brothers with profound intellectual disability, spasticity, congenital heart disease, and unique brain abnormalities [72]. Utilizing WES, novel compound heterozygous mutations in *MED23* were identified, marking a significant discovery in the study of non-consanguineous familial intellectual disability. The radiological examination revealed distinct brain abnormalities in the affected individuals: MRI findings included mild-to-moderate pontine hypoplasia, more pronounced in the older sibling, and mild thinning of the corpus callosum in the younger. Moreover, hypomyelination was evident, particularly affecting the posterior and temporal white matter regions, which suggested altered myelination patterns over time. A. C. Lionel next discusses a 7-year-old boy with refractory epilepsy linked to a novel homozygous pathogenic variant in *MED23* [73]. Born to closely related parents, the child experienced global developmental delay and intractable epilepsy from an early age. Brain MRI revealed delayed myelination and a thin corpus callosum. MRI spectroscopy was normal. WES uncovered a novel homozygous pathogenic variant (c.1937A>G; p.Gln646Arg) in *MED23*, a gene crucial for energy homeostasis and glucose production. A ketogenic diet has proven remarkably effective, eliminating seizures from day one. This case broadens the phenotypic spectrum associated with *MED23* mutations, underlining the potential of the ketogenic diet in this specific scenario [73].

## 16. *MED25* (MIM *610197) [74]

**Figure 8:**
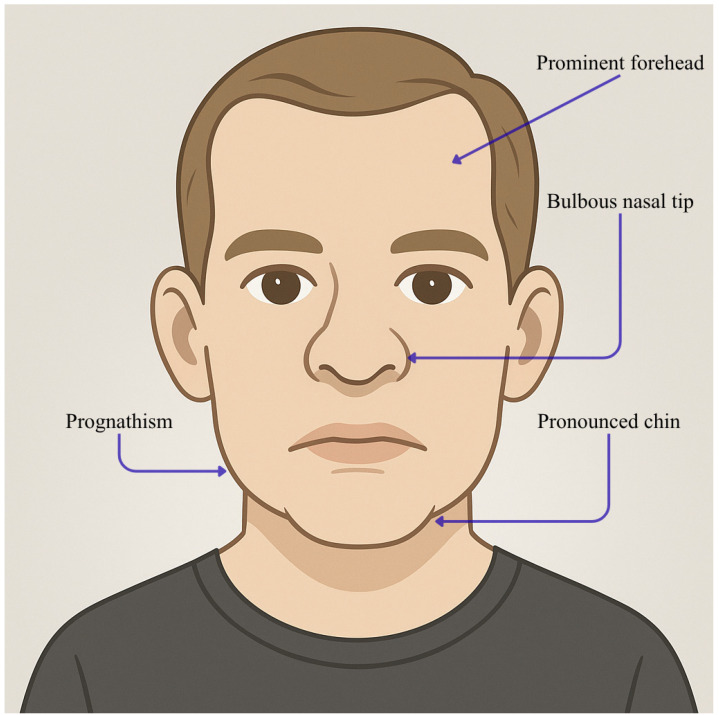
Facies characteristics in *MED25* mutations (c.418C>T; p.Arg140Trp). This figure illustrates an adult male with a prominent forehead, prognathism, a pronounced chin, and a bulbous nasal tip. This image was created using AI, particularly ChatGPT 5. Details regarding the exact procedure are contained in Supplementary Materials.

The *MED25* gene encodes a subunit in the tail of the MED complex, crucial for transcribing most genes dependent on RNA polymerase II. MED25 does not interact directly with RNA pol II but activates or represses transcription by interacting with transcriptional activators (e.g., AP2/ERFs, MYCs, and ARFs), repressors (e.g., JAZs and Aux/IAAs), and other MED subunits (MED13 and MED16) [75]. It is involved in modifying chromatin and forming the pre-initiation complex. Mutations in *MED25* are linked to the pathogenesis of syndromes involving intellectual disability accompanied by eye and brain abnormalities, Warburg micro syndrome (MIM #600118) [76], Kaufman oculo-cerebro-facial syndrome (MIM #244450) [77], Cerebro-oculo-facio-skeletal syndrome (COFS) (MIM #214150) [78], Kahrizi syndrome (MIM #612713) [79], and other syndromes [80]. Lina Basel-Vanagaite et al. demonstrate, using WES, that a *MED25* homozygous mutation (Tyr39Cys) in *MED25* causes a previously unidentified syndrome marked by profound intellectual disability, distinctive dysmorphic traits, congenital eye defects, abnormalities of the corpus callosum and other congenital issues: they assessed seven individuals from four unrelated families living in the same village with WES, clinic valuation and MRI [80]. Facial features include a nevus flammeus simplex on the forehead, receding frontal hairline, sparse hair, sparse eyebrows, transparent skin, epicanthal folds, hypertelorism, short philtrum, wide cupid’s bow, tented upper lip, and everted lower lip vermilion. MRI images show abnormal hyperintense signals in the bilateral occipital periventricular white matter, with a thin corpus callosum noted on midline sagittal images. The clinical and radiological signs seen in the study’s patients overlap with those of other eye–brain syndromes, yet they present a unique and recognizable constellation of features. Shortly after, Figueiredo et al. investigated a consanguineous family in an area of Northeastern Brazil, in which seven adults presented syndromic ID: it was moderate in two members and severe in the remaining five [81]. These individuals, who had not been formally educated and were unable to read or write, shared distinct facial features such as a pronounced forehead, an advanced lower jaw, a noticeable chin, and an unusually large and protruding nose tip. These facial characteristics were absent in both the parents and the five unaffected siblings who underwent clinical evaluation. WES revealed a unique coding variant in *MED25* (c.418C>T, p.Arg140Trp) within the identified linkage regions, presenting it as the only variant potentially responsible for the condition. The facial features described in these patients affected by these mutations in *MED25* were created by AI in our paper and illustrated in Figure 8.

**Figure 9:**
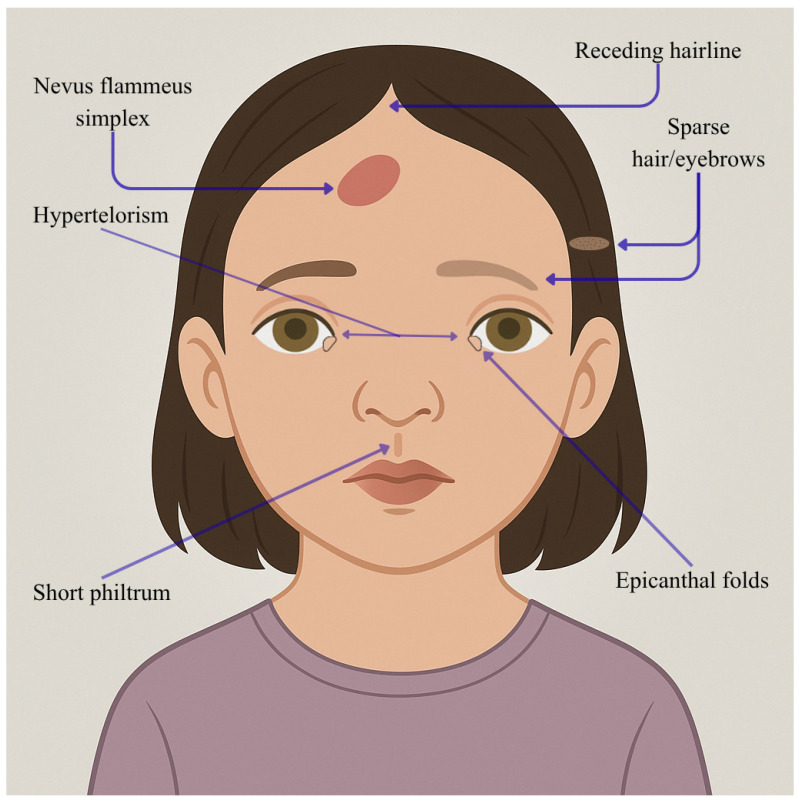
Facies characteristics in *MED25* mutations (Tyr39Cys, homozygous). This figure illustrates a female child (7 years) with a broad forehead, sparse hair and eyebrows, hypertelorism, a short philtrum, a pointed upper lip, an everted lower lip, and a vascular birthmark (nevus flammeus simplex). This image was created using AI, particularly ChatGPT 5. Details regarding the exact procedure are contained in the Supplementary Materials.

Comparing patient populations described by L. Basel-Vanagaite et al. and Figueiredo et al., significant differences in clinical manifestations become evident due to variations in the location and severity of mutations within the VWA domain. Notably, the facial characteristics, such as prognathism, a pronounced chin, and an exceptionally large, protruding nose tip reported by L. Basel-Vanagaite et al., were not observed in the patients from Figueiredo et al.’s study [81]. This absence could be attributed to the age-related development of these features, suggesting a temporal dynamic in their emergence. In contrast, the population studied by L. Basel-Vanagaite et al. [80] displayed distinct eye issues, with 6 of the 7 affected individuals experiencing conditions like cataracts and microcornea. These discrepancies underscore the complex nature of the mutations within the VWA domain and their diverse phenotypic expressions, highlighting the importance of considering both age and mutation specifics in clinical assessments. Another study describes the discovery of the p.A335V mutation in the *MED25* gene among a large family from Costa Rica, showing an inheritance pattern of Charcot-Marie-Tooth (CMT) (MIM #605589) [82] disease [83] that is autosomal recessive, specifically associated with the CMT2B2 region on chromosome 19q13.3. While *MED25* has been linked through functional associations to the activation regions of a variety of transcriptional activators from both cellular and viral origins, its definitive role in modulating transcription remains somewhat elusive. Mutations in *MED25* have been associated with Basel-Vanagaite-Smirin-Yosef syndrome (BVSYS) (MIM #616449) [84], an uncommon autosomal recessive genetic disorder characterized by profound developmental delays and a spectrum of craniofacial, neurological, ocular, and cardiac abnormalities [85]. Specific facial features of these mutations have been illustrated in Figure 9.

I. Maini and colleagues have documented cases involving two sisters afflicted with severe developmental retardation, diminished muscle tone, cardiac malformations, and distinctive facial and limb deformities, which are emblematic of BVSYS [85]. WES facilitated the identification of a homozygous frameshift mutation in the *MED25* gene in the elder sibling. Although this mutation was not verified in the younger sister, the striking concordance in their clinical manifestations strongly suggests its existence. MRI of the brain disclosed anomalies in numerous patients diagnosed with BVSYS, indicating that such complications may be more widespread than previously recognized. Notably, consistently identified features among the siblings were a thin corpus callosum, enlargement of the lateral ventricles, and bilateral perisylvian polymicrogyria. Clinical manifestations, radiological, and genetic characteristics of literature-reported MED-25 related cases are summarized in Table 5.

## 17. *MED27* (MIM *605044) [86]

*MED27* protein is a subunit of the MED complex, which includes the Head and Tail of the MED complex and interacts with MED29, which in turn interacts with MED14 to link the Tail module with the Head and Middle modules [87]. A recent study, employing exome sequencing, unveiled biallelic mutations in the *MED27* gene, implicated in the etiology of a disease, across 16 individuals from 11 distinct families [88]. These subjects exhibited manifestations of an unrecognized neurodevelopmental disorder. The collective symptomatology encompassed severe intellectual impairment, pervasive developmental delays, axial hypotonia coupled with distal spasticity, dystonic movements, and cerebellar underdevelopment. In particularly severe manifestations, individuals also exhibited seizure episodes and cataracts. The identification of biallelic mutations within the MED27 gene underscores its pivotal role in the normal development of neural architectures, with a particular emphasis on cerebellar function. MRI scans of the brain in individuals carrying mutations in both copies of the *MED27* gene, in this study of 11 participants, revealed a spectrum of abnormalities in the cerebellum and the corpus callosum: cerebellar hypoplasia ranging from mild to severe and variations in the thickness of the corpus callosum among patients from their infancy to adult years. Key observations included slight hypoplasia of the vermis, a normal appearance of the corpus callosum in some cases, progressive shrinking of the cerebellum, and simplification of the cortical gyri in certain individuals. Further findings indicated extreme cerebellum hypoplasia, accompanied by flattening of the pons and reduced myelination. While a mild form of hypoplasia was observed in one adult, another exhibited signs of cerebral atrophy but had a normal cerebellum, highlighting the varied neurological effects of mutations in the *MED27* gene. A recent investigation [89] has detailed the characteristics and imaging findings of 57 subjects across 30 families, all harboring homozygous mutations in *MED27*, depicting a neurodevelopmental condition with a neurodegenerative trajectory. This disorder presents early on, characterized by diverse developmental delays, bilateral cataracts, and motor dysfunctions including cerebellar ataxia, dystonia, and spasticity. These symptoms are primarily attributed to cerebellar atrophy along with varying degrees of pons and basal ganglia involvement. The extent of cognitive and motor deficits ranges from severe to less severe, with some individuals living into adulthood, indicating a wide phenotypic variation both within and among families. The research emphasizes the adverse impact of unstable *MED27* mutations in the general population, proposing that the disease-causing mechanism likely involves a partial functional deficit, especially impacting the C-terminal domain. Brain MRI performed in this study uncovered a broad spectrum of abnormalities, from minor reductions in the basal ganglia size to significant cerebellar atrophy, in comparison to standard references. These images shed light on a range of degenerative changes and increased signal intensities in the caudate and putamen nuclei, alongside notable white matter volume reduction and ventricular expansion. More severe cases displayed evident cerebellar deterioration, pontine underdevelopment, and pronounced signal intensity in the cerebellar cortex and dentate nuclei. Other unique findings were the detection of the “hot cross bun” sign, signaling degeneration in the pontocerebellar pathways, microcephaly with a simplified pattern of cortical gyri, and bilateral shrinking of the olfactory bulbs, illustrating the complex impact of this disorder on brain structure, from subtle changes to profound anomalies. Clinical-radiological analysis indicated that more severe clinical manifestations correlated with extensive cerebellar reduction and a slimmer corpus callosum, highlighting the progressive neurodegenerative aspect of the condition [89].

## 18. *CDK8* (MIM *603184) [90]

The *CDK8* kinase module, a component of the MED complex, plays a crucial role in governing RNA polymerase II-mediated gene transcription. Its function encompasses both inhibitory and activating roles in transcription initiation, through reversible association with three other subunits, including cyclin C, MED12, and MED13. Through its kinase activity, CDK8 facilitates dissociation from the MED complex, thereby enabling transcription activation under stressful conditions, while remaining inactive during normal growth phases [91]. The research led by E. Calpena and his team uncovered eight new heterozygous missense substitutions occurring spontaneously in 12 unrelated subjects [92]. A recurrent mutation (c.185C>T, p.Ser62Leu) was found in five individuals. Assessment of the phenotypes of these 12 subjects, aged between 0.1 and 16.7 years, with CDK8 substitutions revealed a wide range of manifestations: early childhood challenges included hypotonia and developmental delays, often resulting in intellectual disability. Behavioral disorders were prevalent, with many subjects diagnosed with either autism spectrum disorder or attention-deficit hyperactivity disorder. Other common issues included neurological, sensory, cardiac, gastrointestinal, and facial abnormalities. MRI of the brain indicated agenesis or thinning of the corpus callosum in four subjects, one of whom had macrocephaly. Missense mutations in *CDK8* lead to a developmental disorder exhibiting phenotypic resemblance to syndromes linked with mutations in other subunits of the MED complex, suggesting overlap in the mechanisms underlying their pathogenesis [92]. In another study, S. Miyamoto et al. identified a *CDK8* variant (c.185C>T, p.Ser62Leu) in a patient with agenesis of the corpus callosum; this demonstrates the close correlation between *CDK8* mutations and alterations of the corpus callosum [93].

## 19. *CDK19* (MIM *614720) [94]

**Figure 10:**
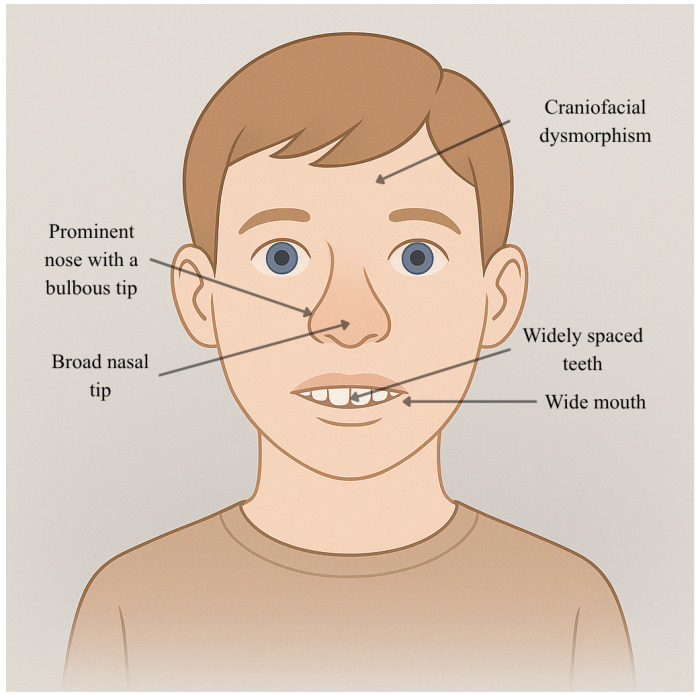
Facies characteristics in CDK19 mutations (heterozygous de novo variants, DEE87). This figure illustrates a male child (7 years) with a prominent nose with a broad and bulbous nasal tip, a wide mouth, and widely spaced teeth. This image was created using AI, particularly ChatGPT 5. Details regarding the exact procedure are contained in Supplementary Materials.

Cyclin-Dependent Kinase 19 (*CDK19*) is a serine/threonine kinase that primarily serves as a transcription regulator, thus, a major contributor to the functions of the nervous system [95]. It is the essential enzymatic part of the Mediator complex’s kinase module, which, by connecting transcription factors to the core RNA Polymerase II apparatus, adjusts gene expression to the required level [95]. In this unit, CDK19 is combined with Cyclin C, MED12L, and MED13L.1 In 2020, it was first evidenced that de novo heterozygous missense variants in *CDK19* cause a new neurodevelopmental disorder, so the disease became known as Developmental and Epileptic Encephalopathy 87 (DEE87; MIM #618916) [96], placing it among the MEDopathies [95]. All damaging mutations are localized to the absolutely conserved serine/threonine kinase domain of the protein; therefore, the changes in enzymatic activity are the main reason for the disease [95]. Most of the mutated variants are found in residues p.Gly28, p.Tyr32, and p.Thr196, resulting in these residues being referred to as mutational hotspots that appear in different patients with genetic disorders [95]. The extent of this revelation is supported by the population genetics evidence; *CDK19* is highly selective for both loss-of-function (pLI score = 1.0) and missense (missense Z-score = 3.56) variation, implying that normal function is the most likely outcome of *CDK19* gene alterations [97]. The DEE87 pathophysiology is characterized by dual loss- and gain-of-function mechanisms. Work with Drosophila models initially led to the conclusion that the human CDK19 protein was functionally identical to its fly homolog CDK8 and, consequently, a loss-of-function (LoF) mechanism was implicated. Patient-derived variants (p.Tyr32His and p.Thr196Ala) failed to rescue the lethal and neurological phenotypes caused by the loss of Cdk8, behaving as strong LoF alleles in this context [95]. However, subsequent research by Zarate et al. (2021) using direct biochemical assays revealed a more nuanced picture [97]. While some variants like p.Gly28Arg did show decreased kinase activity (hypomorphic/LoF), the p.Tyr32His variant exhibited significantly increased kinase activity, classifying it as a gain-of-function (GoF) variant [97]. Experiments in zebrafish embryos confirmed that both LoF and GoF variants were developmentally toxic [97]. This evidence reframes DEE87 as a disorder of kinase dysregulation, where normal neurodevelopment requires CDK19 activity to be maintained within a narrow homeostatic range. Any significant deviation is pathogenic, leading to a convergent clinical phenotype. DEE87 presents as a severe and consistent syndromic neurodevelopmental disorder. The core clinical features are Global Developmental Delay (GDD) and Intellectual Disability (ID) related with expressive language severely impaired, with many individuals remaining nonverbal1; hypotonia, present in a majority of individuals (79%) from infancy and contributing to significant motor delays3; epilepsy, that affect most individuals (64–100%) and are often medically refractor but in some reported cases, other therapeutic modalities such as the ketogenic diet and adrenocorticotropic hormone (ACTH) have also been ineffective in achieving seizure control. The most common presentation is infantile spasms with a hypsarrhythmic EEG pattern, typically with onset between 4 and 15 months of age [95,98]; craniofacial dysmorphism, including a prominent nose with a bulbous tip, a wide mouth, and widely spaced teeth [95]. Craniofacial features are shown in Figure 10. Other frequent findings include ophthalmologic anomalies (64%) and autism or autistic traits (56%) [99]. Brain MRI findings in DEE87 are variable and non-specific, with no single pathognomonic feature identified. Reported abnormalities include mild generalized cerebral atrophy, delayed myelination, and widened cerebral gyri [95]. A subsequent report by Sugawara et al. (2020) on a patient with the recurrent p.Tyr32His variant described non-progressive brain atrophy concomitant with white matter abnormalities suggestive of delayed myelination, which persisted on follow-up imaging [98]. The lack of a consistent, major structural brain malformation is significant. This pattern, combined with functional data from Drosophila models showing a loss of synapses, suggests that DEE87 is primarily a “synaptopathy”—a disorder of neuronal connectivity and function at a microscopic level—rather than a defect in large-scale brain architecture [95].

## 20. Conclusions

This review marks a step forward in understanding the intricate interplay between mutations of the MED complex and the clinical-radiological manifestations observed in MEDopathies. Since mutations in MED complex genes have demonstrated particularly important clinical relevance in pediatric populations, manifesting as a broad spectrum of predominantly neurological symptoms, from psychomotor developmental delay and early-onset epileptic encephalopathies to language impairment and intellectual disability, our study sought to clinically characterize and synthesize the phenotypic features of each individual gene mutation in relation to their neuroradiological profiles, given the rarity of these disorders. While our focus has been on elucidating genotype-phenotype correlations within the MED complex, there is a wealth of genetic data awaiting exploration in the context of MEDopathies. Integrating next-generation sequencing technologies with advanced bioinformatics tools will enable the identification of novel disease-associated genes and variants, broadening the spectrum of MEDopathies and enhancing diagnostic accuracy. Furthermore, as highlighted by the progression of brain abnormalities observed in MED-related NDDs, longitudinal neuroimaging studies are crucial for elucidating disease trajectories and underlying pathophysiological mechanisms. The term ‘MEDopathies’ offers a unifying framework for categorizing disorders arising from defects in different MED complex subunits. However, further characterization of the phenotypic spectrum associated with each subunit is warranted to delineate overlapping and distinctive features among MEDopathies. Integrating clinical, genetic, and radiological data in large-scale databases will facilitate the development of predictive models to streamline the diagnostic odyssey for patients with suspected MEDopathies. In conclusion, while this review provides a comprehensive overview of the current state of knowledge regarding MEDopathies, further study is needed to unravel the complexities of these disorders fully.

Relevant results regarding clinical, radiological, and genetic features in MED-related disorders are summarized in Table 6.

## Figures and Tables

**Table 1:** MRI brain finding in *MED11*-related disorder from the cohort of patients from Calì et al. 2022 [20].

Individual	Scan Age	Radiological Findings
**A-II-1**	1 month	Progressive global neurodegeneration and atrophy involving the cerebral and cerebellar hemispheres
**A-II-1**	4 months	Progressive global neurodegeneration and atrophy involving the cerebral and cerebellar hemispheres
**A-II-1**	2 years	Progressive global neurodegeneration and atrophy involving cerebral and cerebellar hemispheres with cerebral dysgyria |
**A-II-1**	Fetus (31 weeks)	Progressive atrophy of intracranial structures with increasing extra-axial spaces, cerebral dysgyria, and marked cerebellar atrophy
**A-II-1**	Fetus (34 weeks)	Progressive atrophy of intracranial structures with increasing extra-axial spaces, cerebral dysgyria, and marked cerebellar atrophy
**B-II-1**	Not reported	Similar features of global brain underdevelopment with cerebral dysgyria and particular cerebellar atrophy
**B-II-2**	Not reported	Similar features of global brain underdevelopment with cerebral dysgyria and cerebellar atrophy
**B-II-1**	Not reported	Similar features of global brain underdevelopment with cerebral dysgyria, underdevelopment of white matter, and cerebellar atrophy

**Table 2:** *MED12*-related X-linked disorders [31].

Condition	Genetic Basis	Clinical Features	Radiological Features	Notable Findings
**FG Syndrome Type 1 (FGS1)** **(MIM #305450) [27]**	Mutation in *MED12*	Intellectual disability, congenital hypotonia, distinctive facial features	Partial or complete agenesis of the corpus callosum	Role of *MED12* in neurodevelopment
**Nonspecific Intellectual Disability (NSID)**	Genetic heterogeneity	Cognitive impairment without specific physical characteristics	Abnormalities of the corpus callosum; ventriculomegaly	Diverse genetic underpinnings
**Lujan Syndrome (LS)** **(MIM #309520) [28]**	Missense mutation in *MED12* (p.Asn1007Ser)	Intellectual disability, marfanoid habitus, behavioral anomalies	Ventricular dilatation, corpus callosum abnormalities	Impact on brain structure and function
**X-Linked Ohdo Syndrome (XLOS)** **(MIM #300895) [29]**	Missense mutation in *MED12*	Intellectual disability, distinctive facial features, and congenital heart defects	Distinctive changes in brain morphology (Seizures and corpus callosum dysgenesis)	Neurodevelopmental consequences

**Table 3:** Clinical and radiological features of three patients with *Xq12–q13.3* duplication [34].

Features	Patient 1	Patient 2	Patient 3
**Age**	10 Years	4 Years	36 Years
**Motor Development**	Delayed: independent sitting at 15 months, walking at 48 months	Development like patient 1, but more sociable	Slow development, progressive muscle atrophy, periods of agitation
**Expressive Language**	Limited: single words at 7 years, two-word phrases at 10 years	Parallel to Patient 1, but more sociable	Periods of agitation, language decline, and refusal of detailed psychiatric examination
**Physical Anomalies**	Microcephaly, slightly triangular face, flattened nasal bridge, bilateral epicanthic folds, bilateral nicked/crenelated ear lobes, pectus excavatum, partially fused fingers	Similar to Patient 1, but more sociable	Hypertelorism, bilateral cleft of ear lobes, pectus excavatum
**Radiological Findings** **(7 years)**	Mild signal changes bilaterally in dentate nuclei, tegmentum, cerebellar peduncle, and periaqueductal gray matter; mild ventricular enlargement and thinned corpus callosum	Not specified	Thinned corpus callosum, bilaterally enlarged lateral ventricles
**Neurological Examinations (7 years)**	Balance problems, poor muscle tone and strength, and no pathological reflexes	Not specified	Muscle weakness, refusal of a detailed psychiatric examination
**Therapies**	Not specified	Steroids for controlling West syndrome	Not specified

**Table 4:** Summary of MED17-related cases.

Study	Year	Population	MED17 Mutation	Clinical Symptoms	MRI Findings
Kaufman et al. [62]	2010	Israeli children of Caucasian descent	Homozygous missense	Progressive postnatal microcephaly, severe developmental delay	Extensive and pronounced cerebral and cerebellar atrophy, inadequate myelination, diminished thalamic size, and slender brainstem
Agostini et al. [63]	2018	Two adult sisters	Compound heterozygous	Progressive microcephaly, moderate cognitive impairment, epilepsy	Slight cerebellar vermis shrinkage, no cerebral atrophy
Hirabayashi et al. [66]	2019	Two unrelated brothers	Compound heterozygous	Nystagmus, opisthotonic posture, developmental delay, choreiform movements, hypotonia	Slight delays in myelination, anterior horn dilation, and mild cerebellar atrophy
Fattal-Valevski et al. [64]	2020	Unrelated Caucasian-Jewish families	Homozygous	Progressive microcephaly, severe developmental delays, diverse neurological symptoms	Significant cerebral and cerebellar atrophy, reduced white matter volume, delayed myelination, corpus callosum thinning, pronounced ventral pons flattening
Rafiullah et al. [65]	2022	Consanguineous family	Novel homozygous missense	Severe ID, seizures, progressive microcephaly	Mild atrophy, myelination defects

**Table 5:** Summary of *MED25*-related cases.

Study	Mutation	Clinical Manifestations	MRI Findings
Lina Basel-Vanagaite et al. [80]	Tyr39Cys in *MED25*	Profound intellectual disability, distinctive dysmorphic traits, congenital eye defects, and abnormalities of the corpus callosum	Abnormal hyperintense signals in the bilateral occipital periventricular white matter, thin corpus callosum
Figueiredo et al. [81]	c.418C>T, p.Arg140Trp in *MED25*	Syndromic intellectual disability, distinct facial features not seen in parents or unaffected siblings	Not specified
I. Maini et al. [85]	Homozygous frameshift mutation in *MED25*	Severe developmental retardation, diminished muscle tone, cardiac malformations, distinctive facial and limb deformities	Thin corpus callosum, enlargement of the lateral ventricles, bilateral perisylvian polymicrogyria

**Table 6:** Summary of genetic, clinical, and radiological correlations in MEDopathies.

MED Subunit	MIM	Molecular Mechanism	Chromosomal Location	Clinical Features	MRI Findings	Key Points
MED1	*604311	Gene/protein downregulation	17q12	Embryonic lethality Neural tube abnormalities Resistance to breast cancer metastasis	-	Role in growth and differentiation.
MED8	*607956	*SZT2* gene regulation	1q23.1	-	-	Neurological diseases; possible role in epileptogenesis.
MED11	*612383	Homozygous truncating mutation	17p13.2	Neurodegenerative disorder Congenital microcephaly Respiratory distress	Neurodegeneration and atrophy Cerebral dysgyria	Neurodegenerative disorder.
MED12	*300188	Gene missense and duplication mutations	Xq13.1	Intellectual disability; hypotonia; macrocephaly; hyperactivity.	Abnormalities of the corpus callosum; ventriculomegaly	Neurodevelopmental disorders; autism disorders.
MED12L	*611318	Deletions, duplications, nonsense mutations, frameshifts, and splicing mutations	3q25.1	Intellectual disability; developmental delay; attention deficit with hyperactivity; sleep disorders; hypotonia.	Corpus callosum abnormalities.	Autism disorders, attention deficit hyperactivity disorder
MED13	*603808	Truncating, missense mutations, and a single amino acid deletion	17q23.2	Intellectual and developmental delays, language impairments, focal epileptic crisis, and infantile spasms.	Frontal atrophy; dysmorphic corpus callosum.	Developmental and epileptic encephalopathies
MED13L	*608771	Translocations, deletions, and copy number mutations	12q24.21	Intellectual disability; cardiac defects; distinctive facial features	White matter anomalies; abnormalities of the corpus callosum; ventricular dilatation	Pierre Robin’s syndrome; transposition of the Great vessels
MED14	*300182	Unknown mutations	Xp11.4	-	-	Alteration in neural crest cell differentiation
MED 16	*604062	Homozygous truncating variant (novel)	19p13.3	Global developmental delay Severe intellectual disability Congenital heart diseases (Fallot’s Tetralogy) Epilepsy (rare)	Corpus callosum abnormalities Ventriculomegaly	Novel MEDopathy linking MED16 to severe neurodevelopmental disorder
MED17	*603810	Homozygous and heterozygous mutation	11q21	Microcephaly; intellectual disability; epilepsy; ataxia	Cerebral and cerebellar atrophy; inadequate myelination; corpus callosum thinning.	Severe developmental delays.
MED20	*612915	Gene mutations	17q22	Spasticity; dystonia	Cerebral and cerebellar atrophy; basal ganglia hyperintensity	Neurodegenerative movement disorder.
MED23	*605042	Homozygous and heterozygous mutation	6p21.1	Intellectual disability	Pontine hypoplasia; corpus callosum abnormalities; hypomyelination	Epilepsy; developmental delays.
MED25	*610197	- Tyr39Cys mutation; - c.418C>T, p.Arg140Trp mutation; - homozygous frameshift mutation	19q13.33	Intellectual disability; eye abnormalities; congenital defects.	Abnormal hyperintense signals in bilateral occipital periventricular white matter, abnormalities of the corpus callosum	Multiple syndromes involving intellectual disability and eye/brain abnormalities; Charcot-Marie-Tooth disease [82]
MED27	*605044	Biallelic gene mutations	9q34.13	Intellectual impairment; developmental delays; axial hypotonia; distal spasticity; dystonic movements	Abnormalities of cerebellum and corpus callosum; simplification of the cortical gyri; “hot cross bun” sign	Neurodevelopmental and neurodegenerative disorders.
CDK8	*603184	Missense substitutions	13q12.13	Hypotonia; developmental delays; neurological, sensory, cardiac, gastrointestinal, and facial abnormalities.	Agenesis or thinning of the corpus callosum	Neurodevelopmental disorders overlap with other neurological syndromes
CDK19	*614720	Heterozygous de novo missense variants (hotspots p.Gly28, p.Tyr32, p.Thr196)	6q21	Global developmental delay Severe intellectual disability, often nonverbal, ASD Hypotonia, Epilepsy, Infantile Spasms Ophthalmologic anomalies	Mild generalized cerebral atrophy Delayed myelination Widened gyri Non-progressive white matter changes	Developmental and Epileptic Encephalopathy 87 (DEE87); kinase dysregulation with dual loss- and gain-of-function; likely “synaptopathy”

## Data Availability

This article is a narrative review and does not report new clinical data. All information discussed is derived from previously published studies, which are appropriately cited in the References section. Supplementary Materials include the standardized JSON files used to generate synthetic illustrative images via AI, which are available upon request/in the Supplementary Materials repository.

## Supplementary Materials

The following supporting information can be downloaded at: https://www.mdpi.com/article/10.3390/genes16121444/s1, File S1: MED11 c.325C>T (p.Arg109Ter)—faces (JSON); File S2: MED12 p.Asn1007Ser and related faces (JSON); File S3: MED13 p.Pro327Ser and related faces (JSON); File S4: MED13L Pierre Robin related faces (JSON); File S5: MED16—faces (JSON); File S6: MED25 p.Arg140Trp—faces (JSON); File S7: MED25 Tyr39Cys—faces (JSON); File S8: CDK19—faces (JSON).

## Abbreviations

The following abbreviations are used in this manuscript:

ACTH	Adrenocorticotropic Hormone
ADHD	Attention-deficit Hyperactivity Disorder
AI	Artificial Intelligence
ASD	Autism Spectrum Disorders
BDNF	Brain-Derived Neurotrophic Factor
BVSYS	Basel-Vanaigate-Smirin-Yosef Syndrome
CARS	Childhood Autism Rating Scale
CC	Corpus Callosum
CMT	Charcot-Marie-Tooth
COFS	Cerebro-oculo-facio-skeletal Syndrome
DEE87	Developmental and Epileptic Encephalopathy 87
EEG	Electroencephalography
EMG	Electromyography
ER	Estrogen Receptor
ID	Intellectual Disability
KS	Kabuki Syndrome
LS	Lujan Syndrome
MAE	Myoclonic–Atonic Epilepsy
MED	Mediator Complex
MRI	Magnetic Resonance Imaging
MRS	Magnetic Resonance Spectroscopy
NSID	Nonspecific Intellectual Disability
PRS	Pierre Robin Syndrome
WES	Whole Exome Sequencing
WSG	Whole-genome Sequencing
XLOS	X-linked Ohdo Syndrome

## Appendix A. AI-Generated Illustrations Based on Structured Clinical Descriptions

All illustrations depicting clinical portraits (Figure 3, Figure 4, Figure 5, Figure 6, Figure 7, Figure 8, Figure 9 and Figure 10) were generated through ChatGPT and Gemini using JSON-based parameters and progres-sively refined through iterative chat-guided adjustments to enhance accuracy and clarity. Final stylistic elements were edited in Canva. The JSON files are provided as supplemen-tary material to offer insight into the core generative structure, acknowledging that part of the refinement process occurred through conversational fine-tuning.
